# Utilisation of Dental Services of Older People in Australia: An Economic Explanatory Model Based on Cost and Geographic Location

**DOI:** 10.3390/geriatrics6040102

**Published:** 2021-10-20

**Authors:** Wisam Kamil, Estie Kruger, Marc Tennant

**Affiliations:** Department of Anatomy, Physiology and Human Biology, School of Human Sciences, The University of Western Australia, Perth, WA 6009, Australia; estie.kruger@uwa.edu.au (E.K.); marc.tennant@uwa.edu.au (M.T.)

**Keywords:** explanatory model, ageing population, dental services, geographic information system

## Abstract

The increased percentage of older people retaining their natural dentition was associated with a burden of poor oral health and increased service demands. This study analyses the dental service utilisation of the ageing population in Australia and develops a modelled cost design that estimates the dental expenditure required to cover dental services for the aged population. Using the Australian Census of Population and Housing, ageing population and socioeconomic data were mapped to geographic boundaries and integrated with dental service provision data to estimate a model for the utilisation of dental services. The estimated financial cost of dental services was calculated based on the mean fees as per the Australian Dental Association’s Dental Fees Survey. The utilisation of the services varied considerably across the states and also by type of service, with limited numbers using periodontic services. However, there was an increase in cost for replacement and restorative services (5020 million AUD), most evident in the socioeconomic deprivation areas. In addition, the average dental services utilisation cost increased noticeably in the lower socioeconomic deciles of all regions outside major cities. The geographic maldistribution of older people significantly affects the utilisation of dental services, especially among disadvantaged communities. A predicted cost model of 6385 million AUD would cover the oral health needs of older Australians.

## 1. Introduction

Along with the demographic trend of a rapidly ageing population, there is an increase in the number of older people who retain more teeth [1]. However, this incurs an increased risk of periodontal diseases and tooth morbidity associated with high demands for dental services [2]. In addition, the rates of disabilities among the elderly are increasing and pose a profound challenge to the oral health care system [3]. Previous research supports the association between chronic systemic diseases and oral health [4]; this relationship also adds additional pressure on the need for dental care in the geriatric population [5]. Most dental care services in developed countries are privately funded [6], while the subsidised dental treatments are publicly funded, but for limited numbers of the population in which these services are restricted to basic dental care [7]. Moreover, the aged cohort in Australia experience significant barriers to accessing dental services; it has been reported that 29% of low-income people aged 65 and above avoid seeing the dentist; furthermore, 32% of older Australians without dental insurance coverage delay dental care [8]. On the other hand, the increased rate of frequent dental visits among dentate older people reported in the latest National Health Survey of Adult Oral Health [9] would predict the ongoing need for complex treatment of the existing active dental conditions associated with the retained teeth. To the best of our knowledge, the literature lacks an analysis that estimates the expenditure on dental services for older people in Australia; an economic cost-effective explanatory model for the utilisation of dental services could guide policymakers to deploy financial strategies to meet the oral health needs of the ageing population. Therefore, the aims of this study are to anlyse the dental service utilisation of the ageing population in Australia and develop a modelled cost design that estimates the dental expenditure required to cover dental services for the aged population. 

## 2. Materials and Methods

The study data were accessed from publicly available open-access websites; therefore, an exemption from ethics review (RA/4/20/6185) was obtained from the Human Research Ethics Office at The University of Western Australia. 

### 2.1. Population Data

Regarding population data, only people aged 65 years and older were included. The ageing population data were obtained from the 2016 Australian Census of Population and Housing and categorised by Australian States and Territories: New South Wales (NSW); South Australia (SA); Victoria (Vic); Queensland (Qld); Tasmania (Tas) and Western Australia (WA), the Australian Capital Territory (ACT) and the Northern Territory (NT). Microsoft^®^ Excel for Mac (version 16.54, Microsoft, Seattle, WA, USA) was used for data analysis. Our study included the entire ageing population based on Census data; therefore, no statistical analysis was carried out to determine statistically significant differences, since even minor differences are considered valid and true [10].

### 2.2. Geographic Information Data

Geographic information was obtained from the Australian Bureau of Statistics (ABS) website. The boundaries of Statistical Area Level 1 (SA1) are considered the smallest geographic unit of the census data, with a range of population between 200–800 individuals. The data of these geographic units were combined with the SA1 socioeconomic data and integrated to dental service fees and the demographic data of the ageing cohort using Geographic Information System (GIS). 

### 2.3. Socioeconomic Data

The Socio-Economic Indexes for Areas (SEIFA) summarise the sociodemographic characteristics of Australia’s geographic areas and is comprised of four indices. The most commonly used SEIFA index to represent the indicators of disadvantage of the population within a geographical area is the Index of Relative Socio-economic Disadvantage (IRSD) [11]. The IRSD categorises the most disadvantaged (poorest) areas as decile 1 (which is a segment that represents 10% of the population), while the least disadvantaged (wealthiest) areas are classified as decile 10 [12]. 

### 2.4. Modelled Cost Design

To construct a modelled cost design that estimates the expenditure required to cover the total number of older Australians, data were collected on the utilisation of dental services from previously published studies by the Australian Institute of Health and Welfare (AIHW) [8,13]. The AIHW reports statistical information on health and welfare issues at a national level, contributing to policy makers’ decisions on health promotion across the country. First, the average number of dental visits (2.58) by dentate older people was reported from the 2013 National Dental Telephone Interviews [8]. Second, information on the type of service provision as used in this study was based on data presented in the longitudinal study of Dentists’ Practice Activity over two decades (1983–1984 to 2003–2004) [13]. The mean type of services provided for patients aged 65 and over as included in the last wave of the longitudinal study 2003–2004 were used, as this is considered the latest released data on service provided by the age of the population. The number of dental visits received by people aged 65 and over within a period of twelve months was calculated based on the multiplication of the average number of dental visits of the elderly by the total respective ageing population count for each state and territory. Then the number of dental visits was multiplied by the mean number of each service per visit to obtain the number of visits in the main areas of service following the Australian Schedule of Dental Services [14] (Table 1). These included diagnostic, preventive, periodontics, oral surgery, endodontics, restorative, crown and bridge, prosthodontics, and general services. The orthodontics service has been excluded from the modelled framework design of the study since no previous reported data was available in the literature on this dental service by the ageing population.

The estimated dental fees were calculated based on dental fees charged by Australian general dental practitioners (GDPs) in private practice for selected items of schedule of dental services. The mean fees billed by GDPs were published by the Australian Dental Association (ADA) in its Dental Fees Survey Australia 2017 [15]. The ADA publishes the survey of dental fees annually for documentation purposes; however, there is a variation in the dental fees being charged by GDPs within and between the states [15]. We calculated the proposed cost of each category as the average charged fees of total items that have a high frequency of provision of services in each category published in the ADA report of dental fees. Prior to 2020, the 2017 version reported the highest number of dentists with valid responses. Therefore, we included the highest three percentages of service charged by GDPs in each category presented in the 2017 version (Table 2). However, irrelevant items for the dental treatment of older people have been excluded. We estimated the mean items’ fees in each category based on the mean service fee represented in the Dental Fees 2017 (Supplementary Table S1: The average charged fees of provision of service). The fees were integrated to ageing population by states, territory, IRSD deciles, and remote areas.

Due to restrictions and multiple lockdowns during the COVID-19 pandemic [16], the Dental Fees 2020 version was not included in the cost analysis. The estimated financial cost of dental services was integrated to population demographic and deprivation data using Microsoft^®^ Excel and GIS. 

## 3. Results

### 3.1. The Utilisation of Dental Services by Older People

**Figure 1:**
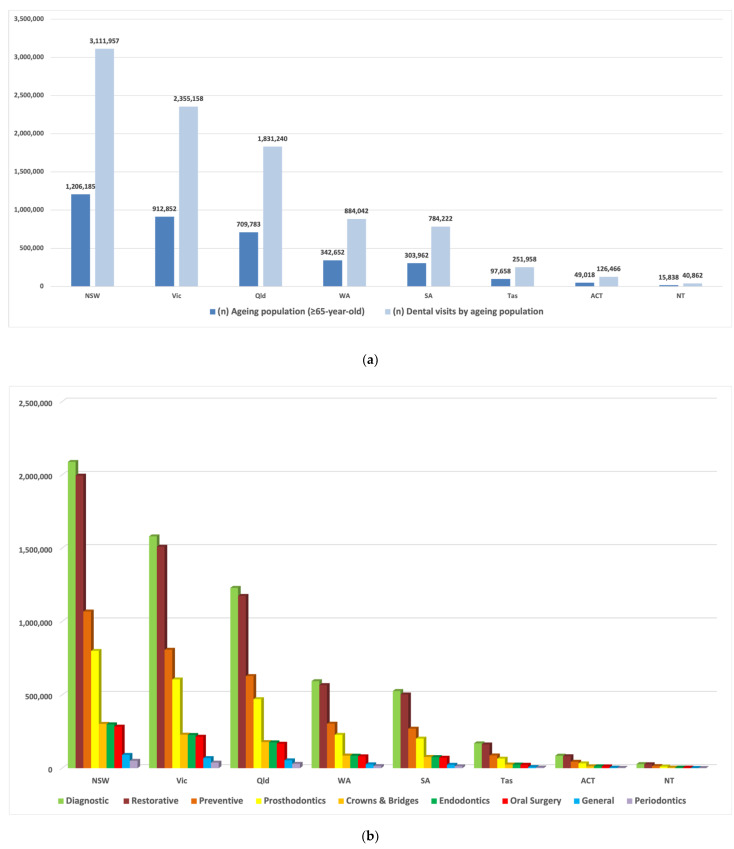
The number of dental visits received by people aged 65 and over, (**a**) The numbers across the states and territories of Australia, (**b**) The numbers by the type of the services. New South Wales (NSW); South Australia (SA); Victoria (Vic); Queensland (Qld); Tasmania (Tas) and Western Australia (WA), the Australian Capital Territory (ACT) and the Northern Territory (NT).

The number of annual dental visits received by people aged 65 and over in the ACT and NT (126,466, 40,862 respectively) and Tas (251,958) account for only up to 10% of total visits presented in other states (NSW and Vic with 3.1 and 2.4 million respectively) (Figure 1a). The descriptive analysis in the main area of service shows that three areas increased in Australia: diagnostic, restorative and preventive, while limited numbers in the service were observed in two main areas, general and periodontics (Figure 1b).

### 3.2. The Estimated Cost of Dental Services 

The rank of dental services by expenditure in Australia parallels the number of visits in four primary service areas, oral surgery, endodontics, general and periodontics, with the lowest value amounted to periodontics as 0.1 and 10 million AUD in NT and NSW, respectively (Table 3). However, the increased cost of replacement and reparative services in prosthodontics, restorative and crown and bridge raised the forecasted spending in these areas across the country, with the maximum expenditure required in prosthodontics 866 million AUD in NSW, with an annual service cost per older person of 1083 AUD compared to only 203 AUD for periodontics. It is worthy to note that using the Department of Veteran’s Affairs (DVA) Schedule [17] alone; the cost calculation would be some 21% lower than in the table. This is not unexpected as the DVA schedule is used as a government price in normal circumstances and not that expected in private practice.

**Figure 2:**
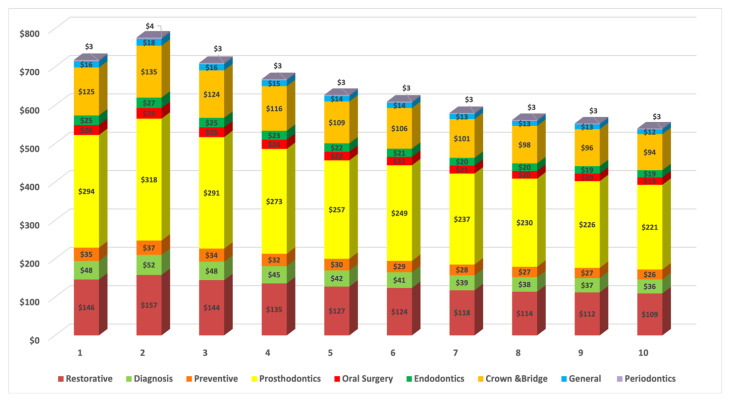
Cost (million Australian dollars, AUD) of dental services utilisation of older population by IRSD, socioeconomic deprivation deciles (1–10). New South Wales (NSW); South Australia (SA); Victoria (Vic); Queensland (Qld); Tasmania (Tas) and Western Australia (WA), the Australian Capital Territory (ACT) and the Northern Territory (NT).

Figure 2 provides detailed estimated costs in the main areas of dental service in the socioeconomic deprivation deciles based on IRSD. The highest cost is predicted in the poorest areas where the older population resides, with 778 million AUD as the highest spent predicted in decile 2. This cost decreased gradually with movement towards the wealthiest areas.

**Figure 3:**
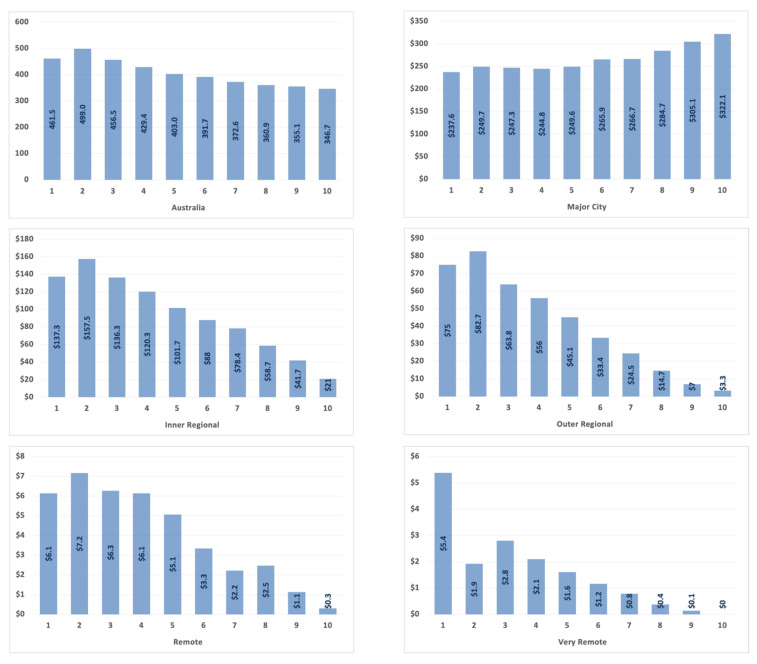
Distribution of the average dental services utilisation cost (million Australian dollars, AUD) of older population by Australia and Remoteness Areas across the IRSD, socioeconomic deprivation deciles (1–10).

The annual average dental service cost for the ageing population by Australia and regional areas across SEIFA index of relative socioeconomic disadvantage deciles was presented in Figure 3. There is an overall trend of increasing dental service costs in Australia’s lowest socioeconomic deciles. However, the difference in the cost becomes evident with the increase in the distance from major cities. These values should be interpreted with caution based on the geographic maldistribution of older people as their demographic decreased significantly away from coastal areas.

## 4. Discussion

It is axiomatic that the total estimated number of dental visits and the type of services received by older people in each state and territory follow the demographic distribution pattern of this population. Therefore, the higher numbers of dental visits identified in this study were in geographical locations where the aging population are concentrated. However, the number of dental visits is expected to exceed those reported in this study, as the percentage of older people in Australia who retained more natural teeth have significantly increased over the past three decades [1]. Hence, the provision of dental services will continue to increase to cover the demands for complex treatment needs. Concerning the type of service provided, the diagnostic service dominates the dental service mix. Diagnosis in dentistry is a daily dental procedure that addresses the patient complaint and determines the provisional treatment plan [18]. Furthermore, the findings of the restorative and preventive services presented in our study may reflect the success in maintaining the natural dentition among the older age cohort over other treatment patterns in the general dental service (extraction, periodontics). Previous national surveys on Australia’s oral health reported increased dental service utilisation among people over 65 [9]. This data justifies the higher number of older people receiving fillings and scaling and the lower number receiving extractions. Although restorative and preventive services become a mainstream approach in the dental treatment philosophy [19], the effect of these predominant services has not demonstrated the significant success in oral disease prevention of the old age cohort [20]. In Australia, oral health reports showed a high prevalence of moderate to severe periodontitis and root decay among older people [21]. On the other hand, research studies of private practice activity, patient characteristics, and service patterns revealed lower dental visits for periodontal treatment [13]. These research studies used a sample based on a longitudinal study design of a random comprehensive sampling frame. However, the reported findings of the average number of periodontic services per visit of people aged 65 and above require careful attention to set a new plan that might yield a slightly different approach to improving the periodontal health of older patients and coping with their treatment needs. The target population in our study included the entire ageing population, therefore sampling error is irrelevant because it involves all persons in the population. However, the lack of individual-level data and confounder information in this area-level population data may be interpreted as a limitation of the study [22].

Improvement in older peoples’ oral health is, among other factors, dependent on access to adequate dental care [23]. Nevertheless, studies have recognised different barriers that affect the utilisation of dental services by older people [24,25]. Geographic location and remoteness have been considered a barrier to delivering oral health care to older people [26]; also, the proximity to capital cities influences the direction of treatment plans and supply of services [27]. Moreover, the maldistribution of the dental workforce and low dentist to population ratio is another evident factor that affects access to dental services [24,28]. Our result indicated Tas with the lowest number of dental visits compared to other states. However, it has the highest percentage of elderly people who live more than 5 km from private and public dental practices and a higher aging population that resides outside of the Greater Capital City areas [29]. The cost of dental treatment is another factor that may impose a barrier against the utilisation of dental services [25]. In Australia, 22.6% of dentate adults reported that the cost prevented them from having a recommended dental treatment [9]. In addition, although dental insurance has been shown to reduce the likelihood of avoiding dental visits by older people [2], about half of older Australians do not have dental insurance [9]. Regrettably, the total national expenditure on dental care of the ageing population by IRSD is the highest in the most socially disadvantaged areas, where most dental services are needed among the elderly residing in these deprived areas. This scenario is quite revealing in the regional and remote areas where disadvantaged areas manifest high-cost oral health demands. In Australia, about one third of people aged 60 and older were reported to have an income less than half the country’s median income, indicating an old age poverty rate [30]. This poor income security, combined with limited access to transportation and mobility restrictions among rural ageing communities, precipitates the effect of increased dental cost [25]. Therefore, the planned financial capacity of our proposed cost was based on the demographic distribution and estimated dental service type of people aged 65 and above in Australia, assuming all older Australians get a fair deal. Research studies and oral health reports advocate for incentive and subsidised payment methods to fill the gaps in the dental health service [31,32]. In Australia, the state-level public dental sectors provide dental services for pensioner cardholders [26]; however, long waiting lists, limited treatment options, and shortages in the dental workforce proposed private dentist reimbursements as an alternative approach, yet momentary limits may add additional obstacles to service provision [7]. We agree that the absence of alternative incentive payment methods like the value-based oral health care approach [33] in our model is a limitation of the study; however, a value-based payment approach priorities disease prevention and oral health promotion rather than defining values reflected by oral health need [33,34]. Value-based payment incentives may be applicable as a national plan that we recommend for future research studies, but it may add no prompt solution to current geriatric oral health issues. Moreover, providing universal access to geriatric dental services requires a high level of coordination between federal and state governments and stakeholders, which is beyond the scope of this paper. Nevertheless, our model can serve as a cost basis for guiding oral health resources and planners of dental services to older people to purchase dental services for the provision of contracted dental services by private dentists. 

Our economic model of oral health expenditure reflected an increase in the cost of prosthodontic services. Unfortunately, the cost of this service is predominant in the dental service mix, despite the consistent decrease in edentulism. The cumulative exposure to oral diseases among older people increases the risk of tooth morbidity, increasing the demand for prosthodontic replacement options to maintain proper oral function and to improve the oral quality of life [35]. However, the descriptive analysis shows a low number of crown and bridge dental visits based on the ageing cohort. The increased cost of this service may reflect a financial burden to accept this expensive delivery pathway of oral care, as the estimated cost points to greater expenditures being required for the repair and replacement of teeth compared to other services. Also, the dilemma posed by the chronic nature of oral diseases among ageing patients affects the utilisation of more complex geriatric dental treatments like tooth/implant-supported crowns and bridges [35]. Tied to this is the amount of remaining bone quality and quantity that leaves the patients as poor candidates for dental implant prosthesis [36], especially among those with impaired health status. In addition, the lack of dexterity and mental deterioration of frail older adults negatively affect oral hygiene practice, where optimal plaque control is a prerequisite in such treatment modalities [37]. Thus, the physiologic and pathologic ageing of the elderly combined with the increased cost of specific indirect restorative and prosthodontic treatments could guide the treatment plan towards use of removable replacement appliances [36,38,39]. Since the burden of periodontal diseases and root caries in the ageing patient remains high [40], geriatric dental research recommends approachable preventive measures for older people [41]. However, the recorded high cost in the prosthodontics service requires more efforts in this particular service to address the community-dwelling needs where a socio-economic gradient exists. Therefore, we recommend an innovative federal government approach to allocate funds for the provision of prosthodontics services for older people experiencing the most barriers to pursue dental services as identified in the study analysis. We are not underestimating the oral health and preventive measures required for this cohort; however, engaging allied dental practitioners, like oral health therapists, to provide essential dental services at a lower cost could alleviate the financial pressure encountered by the elderly. Oral health therapists in Australia are competent to provide non-surgical periodontal and minimal intervention treatments to older patients [42], particularly at early stages of disease severity, to prevent rapid geriatric oral health deterioration. On the other hand, subcontracting public dental services to specialist prosthodontics private clinics may be considered a temporary solution for the oral rehabilitation of ageing patients until the transformation towards a universal scheme for basic dental service access becomes achievable. Additionally, more recognition of geriatric dentistry is required through providing career opportunities in the dental care of ageing patients in special needs clinics and hospitals. Another limitation in this study is that the study population did not identify subgroups of older people, including the Aboriginal or Torres Strait Islander and institutionalised older people, both of which are recognised as having poor oral health [43,44]. Therefore, further research is needed to design an efficient oral health cost model for these subgroups of the older population.

For a decade, dental services expenditure in Australia constituted 6% of recurrent health expenditures, with an average annual growth of 4.4% [45]. However, the Australian Government contributes only $2.4 billion; on the other hand, the non-government contribution ($8 billion) is mainly on a fee-for-service basis by individuals, while 25% is covered through health insurance benefits [45]. It has been reported that 80% of older Australians aged 65 and over depend on the age pension [46]. This reduced income affects the affordability of out-of-pocket expenses and leads to a policy debate [26] that requires the attention of economists and policy makers. The proposed cost (Table 3) estimates the total dental expenditure to reach 6.4 billion; however, the uneven distribution of this cohort across Australia outlines the most deprived areas where older residents most in need of access to dental service are expected to reside. The estimated fees proposed in this study have not considered the differences between the fees charged in each state and territory, as the mean fees were applied; this limitation in the study would also affect the data of services billed for older people in each state and territory, especially in areas of lower socioeconomic status.

## 5. Conclusions

Our estimated model covers a wide range of dental service costs concerning the demographics of the aging population in Australia. It is critical for policymakers and the government to provide a safety net to maintain and promote good oral health and oral quality of life for the ageing population in Australia. This model did not only estimate the fees of dental services utilization, but also exposed the deficiency of the use of specific dental services. Australian baby boomers require a detailed plan to facilitate the utilisation of specific dental services where cost acts as a barrier, particularly for disadvantaged older communities.

## Figures and Tables

**Table 1:** The calculation method to obtain dental visits number (*n*) of people ≥65 by type of dental service in state/territory.

Dental visits (*n*) of people ≥65 within 12 months	=	Mean dental visits of people ≥65	x	People ≥65 (*n*) state/territory	=	Total
Example: Dental visits (*n*) of people ≥65 within 12 months in WA	=	Mean dental visits of people ≥65: 2.58	x	People ≥65 (*n*) in WA: 342,652	=	884,042
Dental visits (*n*) of people ≥65 by type of dental service in state/territory	=	Dental visits (*n*) of people ≥65 within 12 months	x	Mean number of each service per visit	=	Total
Example: Dental visits (*n*) of people ≥65 by type of dental service in WA	=	884,042	x	Diagnostic: (0.671)	=	593,192.182
				Restorative: (0.641)		566,670.922
				Preventive: (0.343)		303,226.406
				Prosthodontics: (0.257)		227,198.794
				Crowns & Bridges: (0.097)		85,752.074
				Endodontics: (0.096)		84,868.032
				Oral Surgery: (0.091)		80,447.822
				General: (0.029)		25,637.218
				Periodontics: (0.016)		14,144.672

**Table 2:** The calculation method to obtain the proposed cost in the main area of service.

Proposed cost in main area of service	=	The average charged fees of total items that have a high frequency of provision of services in each category published in the ADA Dental Fees 2017 report				
The average charged fees of provision of service	=	High frequency of provision of services in the Dental Fees, 2017 (highest three percentages) x				Mean service fee represented in the Dental Fees 2017
Example, proposed cost in diagnostic services:						
		Australian Schedule of Dental Services item numbers				
		Diagnostic Services	000	Mean service fee	Frequency of item billed	Total
		Examinations:				
		Comprehensive oral examination	011	55.20	1	55.20
		Periodic oral examination	012	45.85	1	45.85
		Oral examination—limited	013	28.00	1	28.00
		Radiological Examination, Analysis, and Interpretation:				
		Intraoral periapical or bitewing radiograph—per exposure	022	38.80	2	77.60
Average cost (000)						51.66

**Table 3:** Cost (million Australian dollars, AUD) of dental services of older population by states and territories.

	NSW	Vic	Qld	WA	SA	Tas	ACT	NT	Total
Prosthodontics	$866	$656	$510	$246	$218	$70	$35	$11	$2612
Restorative	$429	$325	$253	$122	$108	$35	$17	$6	$1295
Crown &Bridge	$369	$279	$217	$105	$93	$30	$15	$5	$1112
Diagnosis	$143	$108	$84	$40	$36	$12	$6	$2	$430
Preventive	$102	$77	$60	$29	$26	$8	$4	$1	$308
Oral Surgery	$76	$57	$45	$22	$19	$6	$3	$1	$228
Endodontics	$74	$56	$44	$21	$19	$6	$3	$1	$223
General	$48	$36	$28	$14	$12	$4	$2	$1	$145
Periodontics	$10	$8	$6	$3	$3	$1	$0.4	$0.1	$30

New South Wales (NSW); South Australia (SA); Victoria (Vic); Queensland (Qld); Tasmania (Tas) and Western Australia (WA), the Australian Capital Territory (ACT) and the Northern Territory (NT).

## Data Availability

The data were accessed from publicly available open-access websites; therefore, data availability sharing is not applicable.

## Supplementary Materials

The following are available online at www.mdpi.com/article/10.3390/geriatrics6040102/s1, Table S1: The average charged fees of provision of service.

## References

[1] Hopcraft M.S. (2015). Dental demographics and metrics of oral diseases in the ageing Australian population. Aust. Dent. J..

[2] Australian Research Centre for Population Oral Health (2010). Dental visiting and use of dental services among the Australian older population. Aust. Dent. J..

[3] Cheng X., Yang Y., Schwebel D.C., Liu Z., Li L., Cheng P., Ning P., Hu G. (2020). Population ageing and mortality during 1990–2017: A global decomposition analysis. PLoS Med..

[4] Eberhard J. (2019). General health benefits from good oral health. Aust. Dent. J..

[5] Yellowitz J.A., Schneiderman M.T. (2014). Elder’s Oral Health Crisis. J. Evid. Based Dent. Pract..

[6] Holm-Pederson P., Vigild M., Nitschke I., Berkey D.B. (2005). Dental Care for Aging Populations in Denmark, Sweden, Norway, United Kingdom, and Germany. J. Dent. Educ..

[7] Dudko Y., Kruger E., Tennant M. (2016). National dental waitlists: What would it take to reset to zero?. Aust. Health Rev..

[8] Australian Institute of Health and Welfare 2018 Older Australia at a Glance. Cat. No. AGE 87. Canberra: AIHW. https://www.aihw.gov.au/reports/older-people/older-australia-at-a-glance.

[9] Chrisopoulos S., Luzzi L., Ellershaw A. (2019). Dental care 97. ARCPOH. Australia’s Oral Health. National Study of Adult Oral Health 2017–2018.

[10] Hair J.F., Black W.C., Babin B.J., Anderson R.E., Tatham R.L. (2006). Multivariate Data Analysis.

[11] Wise P., Mathews R. (2011). Socio-Economic Indexes for Areas: Getting a Handle on Individual Diversity Within Areas.

[12] Graham B., Tennant M., Shiikha Y., Kruger E. (2019). Distribution of Australian private dental practices: Contributing underlining sociodemographics in the maldistribution of the dental workforce. Aust. J. Prim. Health..

[13] Brennan D.S., Spencer A.J., Practice Activity Patterns of Dentists in Australia (2006). AIHW Cat. No. DEN 148. Canberra: Australian Institute of Health and Welfare (Dental Statistics and Research Series No. 32). https://www.aihw.gov.au/getmedia/2099a3c3-0154-45a1-9dfe-70a693a5f027/den-148-2000-practice-activity-trends.pdf.aspx?inline=true.

[14] Australian Dental Association (2017). Australian Schedule of Dental Services and Glossary. https://www.ada.org.au/Dental-Professionals/Publications/Schedule-and-Glossary/The-Australian-Schedule-of-Dental-Services-and-(1)/Australian_Schedule_and_Dental_Glossary_2015_FA2_W.aspx.

[15] Australian Dental Association (2017). Dental Practice Surveys—Private Practice Members. https://www.ada.org.au/Professional-Information/Benchmarking/Dental-Fees/Dental-Fees-Survey-Private-Practice-Members-2017/ADA-2017ADADentalFeesSurveyReport_01122017.aspx.

[16] Hopcraft M., Farmer G. (2021). Impact of COVID-19 on the provision of paediatric dental care: Analysis of the Australian Child Dental Benefits Schedule. Community Dent. Oral Epidemiol..

[17] Australian Government (2021). Department of Veterans’ Affairs. Fee Schedule of Dental Services of Dentists and Dental Specialists. https://www.dva.gov.au/sites/default/files/files/providers/feesschedules/dental-fee-sched-jan21.pdf.

[18] Pretty I.A., Maupomé G. (2004). A closer look at diagnosis in clinical dental practice: Part 1. Reliability, validity, specificity and sensitivity of diagnostic procedures. J. Can. Dent. Assoc..

[19] Brennan D.S., Balasubramanian M., Spencer A.J. (2016). Diagnostic services in Australia: Service rates and characteristics of patients. Aust. Dent. J..

[20] Australian Institute of Health and Welfare 2007 Older Australia at a Glance: 4th edition. Cat. No. AGE 52. Canberra: AIHW. https://www.aihw.gov.au/getmedia/ce13dbbe-542c-4957-849c-15109e6a69e7/oag04.pdf.aspx?inline=true.

[21] Do L.G., Luzzi L. (2019). Oral health status 38. ARCPOH. Australia’s Oral Health. National Study of Adult Oral Health 2017–2018.

[22] Thygesen L.C., Ersbøll A.K. (2014). When the entire population is the sample: Strengths and limitations in register-based epidemiology. Eur. J. Epidemiol..

[23] Caldwell J.T., Lee H., Cagney K.A. (2017). The Role of Primary Care for the Oral Health of Rural and Urban Older Adults. J. Rural Health.

[24] Dolan T.A., Atchison K., Huynh T.N. (2005). Access to Dental Care among Older Adults in the United States. J. Dent. Educ..

[25] Kiyak H.A., Reichmuth M. (2005). Barriers to and Enablers of Older Adults’ Use of Dental Services. J. Dent. Educ..

[26] Chalmers J.M. (2001). Geriatric oral health issues in Australia. Int. Dent. J..

[27] Dewanto I., Koontongkaew S., Widyanti N. (2020). Characteristics of Dental Services in Rural, Suburban, and Urban Areas upon the Implementation of Indonesia National Health Insurance. Front. Public Health.

[28] Jean G., Kruger E., Tennant M. (2020). The distribution of dentists in Australia Socio-economic profile as an indicator of access to services. Community Dent. Health.

[29] Kamil W., Kruger E., McGeachie J., Jean G., Tennant M. (2021). Distribution of Australian dental practices in relation to the ageing population. Gerodontology.

[30] HelpAge International. 2015 Global Age Watch Index 2015. https://www.helpage.org/global-agewatch/population-ageing-data/country-ageing-data/?country=Australia.

[31] White J.M., Brandon R.G., Mullins J.M., Simmons K.L., Kottek A.M., Mertz E.A. (2020). Tracking oral health in a standardized, evidence-based, prevention-focused dental care system. J. Public Health Dent..

[32] Bennett C.C. (2009). A healthier future for all Australians: An overview of the final report of the National Health and Hospitals Reform Commission. Med. J. Aust..

[33] Riley W., Doherty M., Love K. (2019). A framework for oral health care value-based payment approaches. J. Am. Dent. Assoc..

[34] Elangovan S., Allareddy V. (2019). Value-Based Payment Approaches. J. Am. Dent Assoc..

[35] Allen F. (2019). Pragmatic care for an aging compromised dentition. Aust. Dent. J..

[36] Dudley J. (2015). Implants for the ageing population. Aust. Dent. J..

[37] Foltyn P. (2015). Ageing, dementia and oral health. Aust. Dental J..

[38] Özhayat E.B., Gotfredsen K. (2012). Effect of treatment with fixed and removable dental prostheses. An oral health-related quality of life study. J. Oral. Rehabil..

[39] Friel T., Waia S. (2020). Removable Partial Dentures for Older Adults. Prim. Dent. J..

[40] López R., Smith P.C., Göstemeyer G., Schwendicke F. (2017). Ageing, dental caries and periodontal diseases. J. Clin. Periodontol..

[41] Wright F., Chu S.Y., Milledge K.L., Valdez E., Law G., Hsu B., Naganathan V., Hirani V., Blyth F.M., Le Couteur D.G. (2018). Oral health of community-dwelling older Australian men: The Concord Health and Ageing in Men Project (CHAMP). Aust. Dent. J..

[42] Teusner D.N., Amarasena N., Satur J., Chrisopoulos S., Brennan D.S. (2016). Applied scope of practice of oral health therapists, dental hygienists and dental therapists. Aust. Dent. J..

[43] Roberts-Thomson K.F., Spencer A.J., Jamieson L.M. (2008). Oral health of Aboriginal and Torres Strait Islander Australians. Med. J. Aust..

[44] Frenkel H., Harvey I., Newcombe R.G. (2000). Improving oral health in institutionalised elderly people by educating caregivers: A randomised controlled trial. Community Dent. Oral. Epidemiol..

[45] Australian Institute of Health and Welfare 2019 Health expenditure Australia 2017–2018. Health and Welfare Expenditure Series no.65. Cat. no. HWE 77. Canberra: AIHW. https://www.aihw.gov.au/getmedia/91e1dc31-b09a-41a2-bf9f-8deb2a3d7485/aihw-hwe-77-25092019.pdf.aspx.

[46] Australian Human Rights Commission (2014). Face the Facts: Older Australians. https://humanrights.gov.au/sites/default/files/FTFOlderAustralians.pdf.

